# Soil Microorganism Interactions under Biological Fumigations Compared with Chemical Fumigation

**DOI:** 10.3390/microorganisms12102044

**Published:** 2024-10-10

**Authors:** Hui Li, Huali Man, Jia Han, Xixia Jia, Li Wang, Hongyu Yang, Guiying Shi

**Affiliations:** 1 College of Horticulture, Gansu Agricultural University, Silver Beach Road Street, Lanzhou 730070, China; lh1767788979@163.com (H.L.); 18794753558@163.com (H.M.); hanjia19991001@163.com (J.H.); 17752210335@163.com (L.W.); yanghongyu3969@gsau.edu.cn (H.Y.); 2 Lanzhou New District Modern Agricultural Development Research Institute Co., Lanzhou 730070, China; 742456030@163.com

**Keywords:** consecutive cropping problems, soil productivity, eggplant, chemical fumigation, biological fumigation, fungal and bacterial communities

## Abstract

Background: Biological fumigation, a potential alternative to chemical fumigation, shows a wide range of prospective applications. In this study, we carried out biological fumigation experiments to evaluate its effect on alleviating consecutive cropping problems (CRPs) when compared with chemical fumigation. Methods: We designed five treatments, namely, CR (no treatment), LN (chemical fumigation with lime nitrogen), Ta (fumigation with marigold), Ra (fumigation with radish), and Br (fumigation with mustard), for soils for replanting eggplant and measured the crop’s growth status, soil bacterial and fungal communities, and soil physicochemical properties. Results: The results showed that the Br and Ra treatments formed similar microbial communities, while the Ta treatment formed unique microbial communities. The genera Olpidiomycota and Rozellomycota could be used as indicator species for the transformation process of soil microbial communities after the Br and Ta treatments, respectively. When compared with the CR and LN treatments, the soil’s physicochemical properties were optimized under the Br treatment, and the soil organic matter content increased by 64.26% and 79.22%, respectively. Moreover, under the Br treatment, the soil’s biological properties enhanced the bacterial and fungal alpha diversity, and the saprotrophic fungi increased with the depletion of pathotrophic fungi, while some specific probiotic microorganisms (such as Olpidiomycota, Microascales, *Bacillus*, etc.) were significantly enriched. In contrast, under the Ta treatment, soil nutrient levels decreased and the soil’s biological indices deteriorated, whereas the bacterial diversity decreased and the pathogenic fungi increased. Conclusions: Among these three biological fumigation methods, the Br pre-treatment was the best way to alleviate the crop’s CRPs and may be a good substitute for chemical fumigation in some situations. However, the Ta treatment also had some risks, such as the loss of land quality and reduced productivity.

## 1. Introduction

Soil chemical fumigation is one of the most effective soil management technologies for promoting long-term soil health in vegetable cropping fields [1]. It can control soil-borne diseases, weeds, and nematodes; therefore, it promotes crop yields. However, the long-term use of chemical fumigation will have a negative influence on the soil and the environment. Several chemical fumigants are commonly used, which include methyl bromide, chloropicrin, lime nitrogen, metham sodium, and dazomet. Among them, the use of methyl bromide has been banned in agriculture due to its harmful effects on the ozone layer [2,3], chloropicrin has been banned in the EU due to its carcinogenic effects [4], and metham sodium weakens the mineralization of carbon and nitrogen in soil and stimulates the emissions of greenhouse gasses such as nitrous oxide (N_2_O). In addition, the application of chemical fumigants is greatly affected by the environmental conditions and application methods, and, if it is used improperly, it can easily cause hazards for crops [4]. In recent years, research on biological fumigation technology as an alternative to chemical fumigation has shown promise for a wide range of applications.

Biological fumigation technology is effective at improving soil health by using plants as biological fumigant materials to control the soil’s pathogenic microflora. At present, cruciferous plants (mustard, radish, cabbage, kohlrabi, cauliflower, etc.) are widely used in biological fumigation. Many cruciferous plants contain a large amount of glucosinolate, which will react with intracellular myrosinase to produce substances named isothiocyanate compounds, which are toxic to soil-borne diseases, pathogens, insects, and weeds, and thus disinfect the soil [5]. When compared with chemical fumigation, the biological fumigation method seems simple. The plant that acts as a biological fumigant is cut up and deeply incorporated into the soil to control soil-borne diseases, insects, and weeds through the decomposition of the chemical thioglucoside in the plant to produce bacteriostatic components and improve the safety of agricultural crop production [6]. A large number of studies have reported that biological fumigation with mustard inhibited the occurrence of soil-borne diseases, changed the soil microorganisms’ community structure, and improved soil fertility [7,8,9]; moreover, biological fumigation with radish increased the yield of fruiting vegetables (e.g., pepper, tomato, and eggplant) by 40% [9].

This research was conducted in Northwest China, where the climate is cold and dry and cruciferous plants are widely cultivated as vegetables or cash crops. Therefore, it was necessary to research the value of biological fumigation to disinfect the soil by using cruciferous plants as biological fumigant materials in this area.

Asteraceae (marigold (*Tagetes erecta* L.)) is an important biological fumigation material for which the fumigation mechanism differs from that of cruciferous plants. Marigold contains a variety of microorganism-inhibiting ingredients which have complex functions against pathogenic fungi or bacteria. The literature has shown that the chemical composition of marigold includes thiophenes, essential oils, pigments, flavonoids, and glycosides. Among these, α-trithiophene is the most toxic substance with the highest content [10,11]. It has been reported that marigold treatment can inhibit the occurrence of watermelon wilt disease, promoting watermelon plants’ growth [12], and can control tomato early blight disease [13]. At present, the application of marigold as a biological fumigation material is very limited because its seed is very expensive. However, due to both its flowers and leaves having a high medicinal value [12], it is widely cultivated as a medicinal plant in the region (Gansu Province, Western China, Supplementary Figure S1). After the flowers have been harvested, a large number of marigold plant residues are left in the field and are turned over and buried via plowing during autumn of the same year. Our investigations revealed that the successor crops after the cultivation of marigold, such as beans, melons, and solanaceous vegetables, were severely inhibited according to many local farmers, which meant that the soil’s function might have been damaged by culturing marigold or due to pollution by its plant residues. Therefore, it is very important to evaluate the value of biological fumigation with marigold and its ecological risk in the region.

Eggplant (*Solanum melongena* L.) is a popular vegetable in Asia. Eggplants, peppers, and tomatoes are major vegetables all over the world (especially in China). These three vegetables belong to the solanaceae, which suffer similar diseases and have similar cultivation techniques. Thus, we selected eggplant as a model vegetable to study biological fumigation as an alternative technology to chemical fumigation technology, which is valuable for promoting efficient and low-toxicity soil remediation technology, and which would be helpful for producing this major vegetable with a high quality and safety in China.

In nature, the soil’s biodiversity is positively correlated with sustainable productivity [14], the loss and simplification of which have a negative impact on maintaining a variety of ecosystem functions, including plant diversity, the decomposition of organic matter, nutrient retention and cycling, and so on [15]. As mentioned above, brassica plants and marigolds have different mechanisms for controlling soil-borne diseases, so they must impact the soil’s microbial diversity differently. Many studies have revealed that biological fumigants from brassica and marigold altered the bacterial community’s structure [16] or the fungal community’s structure [17,18] in different crop planting systems. However, fewer studies have compared the community structure of microorganisms (both fungi and bacteria) in the soil between these two biological fumigants in the same crop planting system. We found one study [19] which evaluated apple trees’ growth status via the shoot length and discussed some specific microorganisms that might be related to apple replant disease. In recent years, software for predicting the microorganisms’ function have been developed (e.g., FUNGuild software). This method provides an effective way to understand the mechanisms maintaining the soil’s function. It is necessary and interesting to understand the differences in soil microorganism biodiversity associated with soil function with the assistance of software for predicting the microorganisms’ function. As mentioned above, the application of chemical fumigants is greatly affected by the environment. Our team previously proved that, if chemical fumigants are applied in early spring in Gansu province, which is cold and dry, it easily leads to crop hazards, because the chemical substrates cannot decompose completely in soil with a lower temperature and insufficient moisture, and they will damage plants [4]. Moreover, eggplant is widely grown in Gansu Province, and CRPs occur commonly. The biofumigants marigold, radish, and mustard are also widely grown in this region. Thus, we undertook this research to explore the optimal biological fumigation, which might be an alternative to chemical fumigation. For this study, we proposed the following hypotheses. Biological fumigation will substantially alter the soil’s properties, and the optimal biological fumigation must promote soil health by increasing microbial alpha diversity. Some specific beneficial microorganisms will increase with the depletion of some harmful microorganisms. In addition, many studies have reported marigold as an effective biofumigant [10,11,12], but fewer studies have focused on its negative influence on soil productivity. This research provides much data about the negative influence of marigold crops on the soil’s productivity and contributes some suggestions on recovering the soil’s productivity. Therefore, this study has special novelty and value for areas in Gansu province, where marigold is commonly grown.

Lime nitrogen is a very good alkaline and non-polluting fertilizer. Our research has shown that chemical fumigation with lime nitrogen effectively alleviated eggplant CRPs in a solar greenhouse [20]. In this experiment, we collected soil samples after fumigation treatments, characterized the bacterial and fungal communities’ structure using high-throughput DNA sequencing technology (Miseq), and predicted fungal function by using FUNGuild software (http://218.2.224.234:8888/analysis/toolTask, accessed on 7 April 2023). By combining the results with the eggplants’ growth and yield data, we finally evaluated the soil’s health and production under different treatments. The main objectives of this study were (1) to explore the differences in bacterial and fungal communities’ structures associated with soil function between biological fumigation with cruciferous plants and Asteraceae on replanting soil; (2) to discover if the marigold treatment negatively affected the soil’s functions and, if so, the reason for this damage.

## 2. Materials and Methods

### 2.1. Description of the Field and Collection of Soil Samples

The study site is a field located in Lanzhou City, Central Gansu Province (36°14′38″ N-36°47′29″ N, 103°54′24″ E-104°24′55″ E). It belongs to a temperate continental arid and semi-desert climate, with an average annual temperature of 8.07 °C and an average frost period of 183.8 days. The annual average precipitation is 198 mm, and the annual average evaporation is 1997.1 mm. It is an important agricultural area of Yellow River irrigation in Gansu Province, with sufficient water sources and good agricultural irrigation conditions. The soil is locally known as Huangmian soil (a deep soil layer, high water storage capacity, a pH of 7.8, and 1.3% organic matter).

This study was conducted from March 2018 to September 2021 in three parts. First, we cropped eggplants continuously for 3 years (2018, 2019, and March to May in 2020). Secondly, we carried out the soil fumigation test in 2020 (from May to October) and detected the soil’s physicochemical and microbial properties. Finally, in 2021, we planted eggplants and evaluated their growth status and yield. In addition, our previous study showed that the ratio of the number of cultivable bacteria/the number of cultivable fungi (B/F values) can be used as a reliable indicator for evaluating the soil quality [21], so we determined the number of culturable bacteria and culturable fungi in the soil.

We designed five treatments, namely, CR (no treatment), LN (chemical fumigation with lime nitrogen), Ta (fumigation with marigold), Ra (fumigation with radish), and Br (fumigation with mustard), and set up three replicates of each treatment with a plot size of 10 m^2^ (5 m × 2 m).

The biological fumigation treatment method was based on Bunlong Yim’s study [19], with slight modifications. On 15 May 2020, radish (30 kg/ha), mustard (12 kg/ha), and marigold (10 kg/ha) were ridge-planted (row spacing: 0.30 m × 0.30 m) in a cropping field with eggplants planted continuously for 3 years. The plants were dug up and chopped to a length of about 3–5 cm and buried in the soil to a depth of 30 cm at the full-flowering stage of the plants (15 August 2020 for radish and mustard, and 4 October 2020 for marigold); for the LN treatment, the lime nitrogen granules (total nitrogen ≥20%, calcium cyanamide ≥50%; Ningxia Yuanda Xingbo Chemical Co., Ltd., Yinchuan, China) were evenly sprinkled on the surface (0.1 kg/m^2^), then plowed into the soil at a depth of 30 cm. After this practice, the soils were irrigated with water (45 L/m^2^) and covered with a plastic film for 30 days; then, we removed the film and let it dry for 30 days. In the following year (beginning on 15 May 2021), eggplant seedlings were cultivated with high-ridge mulching (width of ridges, 0.9 m; width of ditches, 0.45 m; height of ridges, 0.25 m; two rows of ridges; plant spacing, 0.45 m) with single-stem pruning. Cultivation was completed on 20 September 2021.

Soil sampling collection: The first soil sample was taken after the completion of the fumigation treatment (20 June 2020) to determine the physicochemical properties and microbial community structure of the soil. A five-point sampling method was used, five soil samples were collected in each plot, and the sampling depth was 20 cm. After sorting with a 1 mm sieve, 20 g soil samples were collected, divided into two parts, and immediately frozen with liquid nitrogen. We placed a portion (10 g) into a centrifuge tube and sent it to a biotech company to determine the soil microbial community’s structure. The other part was used for the determination of the soil’s physicochemical and biological properties. The second sampling was carried out during the growing period of the eggplants (collected at 20 d, 80 d, 110 d, and 140 d after the establishment of the eggplant seedlings) and was used to determine the number of culturable microorganisms. The sampling methods were as described above.

### 2.2. Determination of the Soil’s Physiochemical and Biological Properties

For the soil samples collected after the fumigation treatments (collected on 20 June 2020), eight physical and chemical properties of the soil were measured, including the organic matter, electrical conductivity, pH value, soil water content, urease, sucrase, alkaline phosphatase, and catalase. The organic matter, electrical conductivity, pH value, and soil water content were examined with reference to our published study [22]; the urease, sucrase, alkaline phosphatase, and catalase activities were analyzed with reference to another of our published studies [23].

For the eggplant cultivation period soil, the numbers of culturable bacteria and fungi were determined. The bacteria and fungi were cultured using a beef paste peptone medium and Martin’s medium, respectively, and they were enumerated using the dilution-coated plate method [21].

### 2.3. Determination of Plant Growth and Yield

We randomly selected 10 plants during the fruiting period of the eggplant (17 June 2021) to evaluate their growth status. Four indicators of seedling growth (plant height, stem thickness, and the biomass of the aboveground and belowground parts) were measured. Starting from the first fruit, all of the fruits were harvested from each plot for determining the yield, and the period when the yield was counted was from 18 July to 20 September 2021.

### 2.4. Throughput Sequencing by Illumina Miseq

#### 2.4.1. DNA Extraction

DNA from the different samples was extracted using the E.Z.N.A. ^®^Soil DNA Kit (D4015, Omega, Inc., Norcross, GA, USA) according to the manufacturer’s instructions. The reagent, which was designed to uncover DNA from trace amounts of the sample, was shown to be effective for the preparation of the DNA of most bacteria. Nuclear-free water was used for the blank. The total DNA was eluted in 50 μL of an elution buffer and stored at −80 °C until measurement via PCR by LC-Bio Technology Co., Ltd., Hangzhou, China.

#### 2.4.2. PCR Amplification, ITS Sequencing, and 16S rDNA Sequencing

The ITS2 region of the small-subunit rRNA gene of the fungi was amplified with slightly modified versions of the primers ITS1-F (5′-GTGARTCATCGAATCTTTG-3′) and ITS2-R (5′-TCCTCCGCTTATTGATATGC-3′) [24]. The 16S region of the small-subunit rRNA gene bacteria was amplified with slightly modified versions of the primers 341F (5′-CCTACGGGNGGCWGCAG-3′) and 805R (5′-GACTACHVGGGTATCTAATCC-3′) [25]. The 5′ ends of the primers were tagged with specific barcodes for each sample and universal sequencing primers. PCR amplification was performed in a reaction mixture with a total volume of 25 μL containing 25 ng of the template DNA, 12.5 μL of PCR Premix, 2.5 μL of each primer, and PCR-grade water to adjust the volume. The PCR conditions for amplification consisted of an initial denaturation at 98 °C for 30 s; 32 cycles of denaturation at 98 °C for 10 s, annealing at 54 °C for 30 s, and extension at 72 °C for 45 s; and then a final extension at 72 °C for 10 min.

The PCR products were confirmed via 2% agarose gel electrophoresis. Throughout the DNA extraction process, ultrapure water, instead of a sample solution, was used as a negative control to exclude the possibility of false-positive PCR results. The PCR products were purified with AMPure XT beads (Beckman Coulter Genomics, Danvers, MA, USA) and quantified by Qubit (Invitrogen, Carlsbad, CA, USA). The amplicon pools were prepared for sequencing, and the size and quantity of the amplicon library were assessed on an Agilent 2100 Bioanalyzer (Agilent, Palo Alto, CA, USA) and with the Library Quantification Kit for Illumina (Kapa Biosciences, Woburn, MA, USA), respectively. The libraries were sequenced on a NovaSeq PE250 platform (Illumina, San Diego, CA, USA).

### 2.5. Data Analysis

The data analysis was based on the cloud services of the Beijing Allwegene company (https://www.allwegene.com, accessed on 25 August 2020). The resulting high-quality sequences were extracted and classified according to the minimum sample sequence number before analysis. The effective sequences of the soil samples were clustered into operational taxonomic units (OTUs) with a similarity degree of 97% using Uparse [26]. The Venn software (http://218.2.224.234:8888/analysis/toolTask, accessed on 7 April 2023) [25] used R tools for the statistics and plotting the results of the OTU clustering. The alpha diversity index [27] was calculated on the basis of the OTUs with Mothur software (version 1.45.3). The PCA (principal coordinate analysis) [28] was performed using the R package (version 3.6.0) to examine the statistical significance of the microorganisms’ structural similarity and the soil’s physiochemical properties under different treatments.

To determine the significantly important microbial taxa, a linear discriminant analysis effect size (LEfSe) analysis was carried out as previously reported [28]. The LDA effect size [29] analysis was carried out to detect species with significant differences in abundance at the genus level with groups with different fumigation treatments, with the threshold set at 2.5. To further assess the relationship between the composition of the microorganism community and the physicochemical properties under different soil treatments, a redundancy analysis (RDA) was performed, based on fungal and bacterial abundance at the genus level and physicochemical parameters, using the R package. The fungal functions were predicted with the software tool FUNGuild (Fungi Functional Guild).

Univariate analysis of variance and Duncan’s multiple comparison method were used to detect the significant differences (*p* < 0.05).

### 2.6. Accession Numbers

The sequences were submitted to the NCBI Sequence Read Archive (SRA) under the Bioproject ID PRJNA1139406.

## 3. Results

### 3.1. The Soil’s Physicochemical Properties after the Eggplant Soil Fumigation Treatments

The soil’s physicochemical properties significantly changed after the fumigation treatment (Table 1). Biological fumigation significantly increased the soil organic matter content. Compared with the CR treatment, the organic matter content of the Ra, Br, and Ta treatments increased by 49.11%, 46.26%, and 37.84%, respectively. The Ta treatment showed a significant decrease of 30.30% compared with the LN treatment. The soil water content varied to a lesser extent, and the water content of the Ta treatment decreased by 5.88% compared with the CR treatment. Compared with the CR treatment, the LN and Br treatments significantly reduced the soil’s pH value. Soil enzyme activity is a comprehensive indicator that reflects the level of soil nutrients and their absorption and utilization status. After the fumigation treatment, sucrase activity increased significantly compared with the CR treatment, and both catalase and alkaline phosphatase activity also changed significantly under the different treatments.

### 3.2. Fungal and Bacterial Diversity after the Eggplant Soil Fumigation Treatments

**Figure 1:**
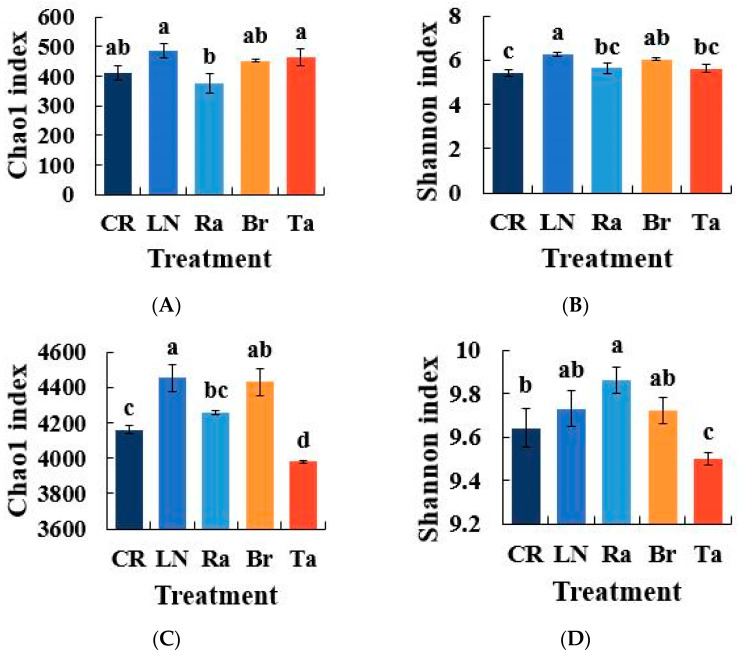
Alpha diversity analysis showing the OTUs after the eggplant soil fumigation treatment, as analyzed by Illumina Miseq ((**A**,**B**) are the Chao1 index and Shannon index of the fungi, and (**C**,**D**) are the Chao1 index and Shannon index of the bacteria, respectively). Different lowercase letters indicate significant differences among the different treatments (*p* < 0.05).

The alpha diversity index was evaluated on the basis of the OTUs. The Chao1 index and Shannon index reflect the richness and diversity of species within a single sample, respectively. Both the biological and chemical fumigation treatments altered the microbial community diversity of fungi and bacteria (*p* < 0.05). For the fungi, the Chao1 index and Shannon index of the LN treatment were generally higher than those of the CR treatment and the three biological treatments (Figure 1A,B). Among the three biological fumigation treatments, the Chao1 index of the Ta treatment was significantly higher than that of the Ra treatment (Figure 1A).

For the bacteria, the Chao1 index and Shannon index of the LN, Ra, and Br treatments were higher than the CR treatment. The Chao1 index and Shannon index of the Ta treatment were the lowest compared with the CR treatment. The Chao1 index decreased by 6.98% compared with the highest value (LN treatment), whereas the Shannon index decreased by 2.23% compared with the highest value (Ra treatment), and the level of bacterial microbial diversity significantly decreased (Figure 1C,D).

**Figure 2:**
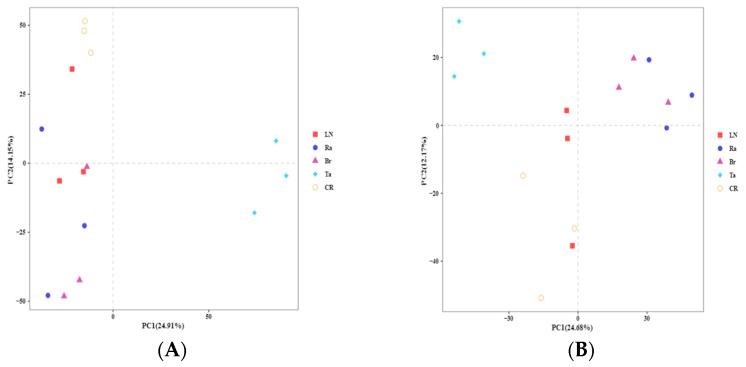
Principal components analysis (PCA) showing overall structural changes in the fungi (**A**) and bacteria (**B**) after the eggplant soil fumigation treatment according to Illumina Miseq.

The results of the beta diversity analysis indicated that the fungi and bacteria in each soil sample were clearly divided into the following two groups: the Ta treatment formed a unique microbial community, while the other four treatments formed a group. Among them, the Br treatment and the Ra treatment had similar microbial communities, while the LN treatment and CR treatment had similar communities. For the fungi, both the first and the second component axes explained 39.06% of the variation (Figure 2A). For the bacteria, both the first and the second component axes explained 36.85% of the variation (Figure 2B).

### 3.3. Overall Structures of Fungi and Bacteria after the Eggplant Soil Fumigation Treatments

**Figure 3:**
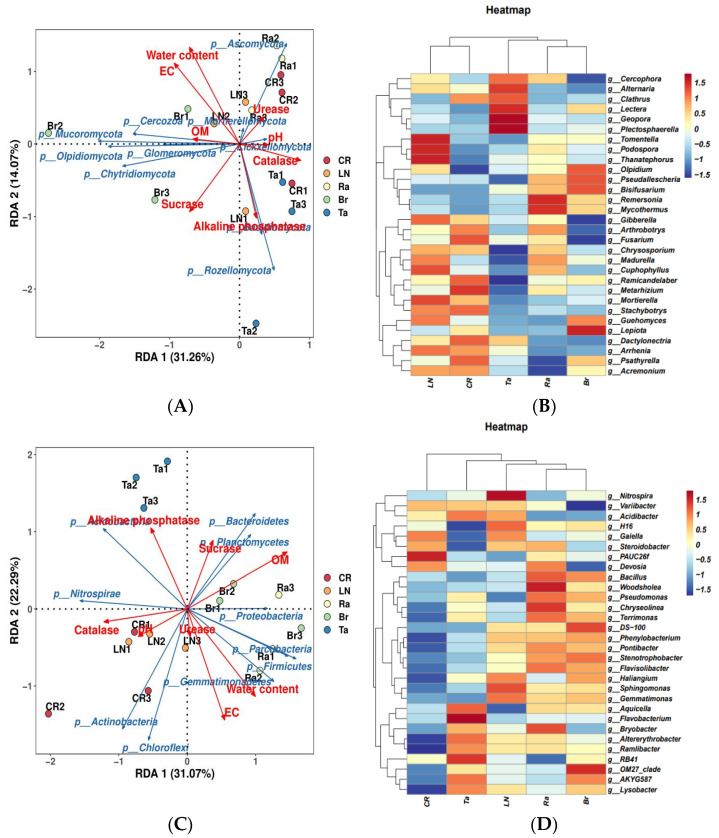
Redundancy analysis (RDA) of fungal community levels and physicochemical properties (**A**) and heat map at the genus level (**B**), and redundancy analysis (RDA) of bacterial community levels and physicochemical properties (**C**) and heat map at the genus level (**D**) after the eggplant soil fumigation treatment. OM and EC represent organic matter and electrical conductivity, respectively.

The RDA showed that the microbial community after the fumigation treatment was significantly influenced by the soil’s physical and chemical properties. Among these, the organic matter content, electrical conductivity, water content, and pH of the soil had a particularly significant impact on the microbial community after the fumigation treatment. Fungi and bacteria explained 45.33% and 53.36% of the variance, respectively (Figure 3A,C).

For the fungi, 14 phyla were identified in total, with the dominant phyla being Ascomycota, Mortierellomycota, Basidiomycota, Rozellomycota, and Olpidiomycota (average RA > 2%). At the genus level, 281 genera were identified in total; the dominant genera were *Mortierella*, *Metarhizium*, *Plectosphaerella*, *Chrysosporium*, *Olpidium*, *Acremonium*, and *Mycothermus* (average RA > 2%) (Figure 3B). For the bacteria, 45 phyla were identified in total, among which the dominant phyla were Bacteroidetes, Chloroflexi, Gemmatimonadetes, Actinobacteria, Acidobacteria, and Proteobacteria (average RA > 5%). At the genus level, 432 genera were identified, of which the three dominant genera were *RB41*, *H16*, and *Sphingomonas* (average RA > 1%) (Figure 3D).

The results of the cluster analysis at the microbial genus level were generally consistent with the results of the PCA. The fungal communities of the LN and CR treatments clustered together, and those of the Ra and Br treatments all clustered together, while those of the Ta treatment clustered alone (Figure 3B). The results of the clustering analysis at the bacterial genus level were partly consistent with the results of the PCA. The bacterial communities under the Ra and Br treatments were clustered together, while the CR treatment was separately classified into one group (Figure 3D).

### 3.4. Specific Microorganism Groups after the Eggplant Soil Fumigation Treatment

The study of the dominant phyla with significant changes in abundance at the phylum level under different treatments (*p* < 0.05, average RA > 1%) revealed that four fungal phyla showed significant changes in abundance among the treatments, with Ascomycota being the most abundant among the five treatments, ranging from 47.75% to 66.99%. Rozellomycota underwent significant changes under the Ta treatment, with a significant increase in relative abundance, which was 20 times higher than the other treatments; Olpidiomycota showed significant changes under the Br treatment, with an abundance 5 times higher than the other treatments. For the bacteria, the phylum-level abundance changed significantly among the treatments, but no bacterial phyla that increased by orders of magnitude were observed to exist. Among the five treatments, Acidobacteria had the highest abundance, accounting for 23.72% to 35.33% (Table 2).

**Figure 4:**
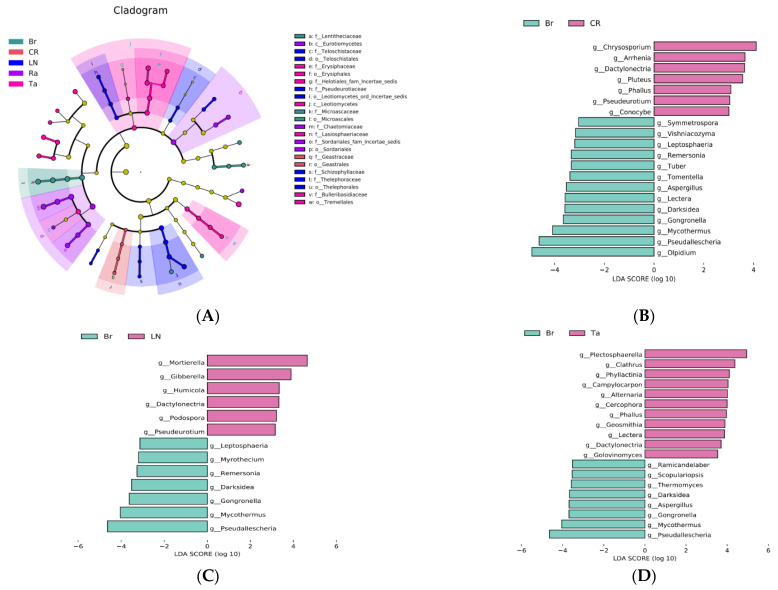
LEfSe analysis showing changes in the dominant fungal communities after the eggplant soil fumigation treatment, generated by Illumina Miseq. Cladogram obtained from the phylum to family levels (**A**); LDA obtained at the genus level between pairs of treatments (**B**–**D**).

The LEfSe analysis further demonstrated the microbial communities and their affiliations that underwent significant changes. The results of the fungal LEfSe analysis showed that, in the CR treatment, Gestrales was significantly enriched; under the LN treatment, Thelephorales was significantly enriched from order to family; under the Ra treatment, Sordariales was significantly enriched; under the Br treatment, Microascales was significantly enriched from order to family; under the Ta treatment, Erysiphales and Tremelales were significantly enriched from order to family (Figure 4A).

**Figure 5:**
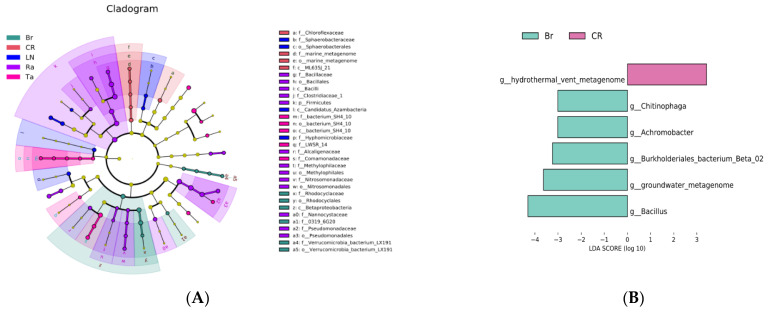

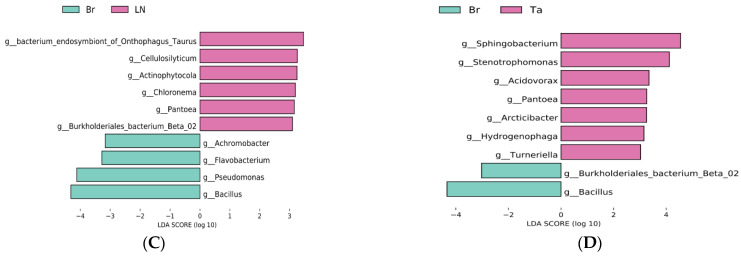
LEfSe analysis showing changes in dominant bacterial communities after the eggplant soil fumigation treatment, generated by Illumina Miseq. Cladogram obtained from the phylum to family levels (**A**); LDA obtained at the genus level between pairs of treatments (**B**–**D**).

The LEfSe analysis results for the bacteria indicated that, in the CR treatment, the order ML635J_21 was significantly enriched; under the LN treatment, the order Sphaerobacterales was significantly enriched from order to family; under the Ra treatment, Firmicutes was the most abundant, and Bacilli and Pseudomonadales were significantly enriched; under the Br treatment, Betaproteobacteria was significantly enriched from order to genus; under the Ta treatment, the order bacterium_SH4_10 was significantly enriched from order to family (Figure 5A).

The results of the analysis above indicated that the Ta treatment formed a unique microbial community structure, while the microbial community structures of the Ra and Br treatments were similar. In order to reveal the specific functional characteristics of the microbial community caused by the biological fumigation treatment with cruciferous material, chemical fumigation treatment, and biological fumigation treatment with marigold, we conducted an LDA at the genus level for the Br and CR, Br and LN, and Br and Ta treatments. The results showed that, for the fungi, compared with the CR treatment, the genus *Olpidium* was significantly enriched under the Br treatment; compared with the LN treatment, *Gibbberella* was significantly enriched under the Br treatment; compared with the Br treatment, *Alternaria* was significantly enriched under the Ta treatment (Figure 4B–D). For the bacteria, the relative abundance of *Bacillus* increased significantly under the Br treatment compared with the CR, LN, and Ta treatments. In addition, compared with the LN treatment, *Pseudomonas* significantly increased under the Br treatment (Figure 5B–D).

### 3.5. Functional Prediction of Fungi after the Eggplant Soil Fumigation Treatments

**Figure 6:**
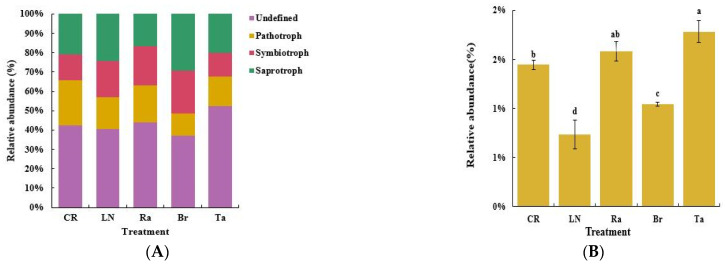
Functional predictions of soil fungi after the soil fumigation treatments: three basic trophic types (**A**) and plant pathogens (**B**). Different lowercase letters indicate significant differences among the different treatments (*p* < 0.05).

Functional prediction of the fungi was carried out with FUNGuild online analysis software. The results showed that the three basic nutrient modes of soil microbes (pathotrophs, symbiotrophs, and saprotrophs) were represented differently in each treatment. Among them, the proportion of saprotroph fungi under the Br treatment was the highest, and these fungi exchange nutrients by degrading dead host cells. Pathotrophs, fungi that obtain nutrients by damaging the host cells, decreased by 28.5% under the LN treatment and by 50.08% under the Br treatment compared with the CR treatment (Figure 6A). On the basis of the three nutrient modes listed above, the fungi were subdivided into 18 guilds with specific functional roles (Supplementary Figure S2). Among the pathotrophs, plant pathogens were the most abundant under the Ta treatment, the second most abundant under the Br treatment, and the least abundant under the LN treatment (Figure 6B).

### 3.6. Evaluation of Eggplant’s Growth Status and Yields Combined with the Soil’s Biological Characteristics during Crop Cultivation under Different Soil Fumigation Treatments

**Figure 7:**
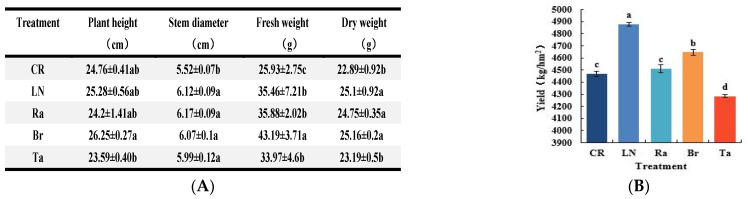
Eggplant’s growth status (**A**) and yield (**B**) during the growth period after different soil fumigation treatments. The date of measurement of the plant growth index was 17 June 2021. The yield was measured from 18 July 2021 to 20 September 2021. Different lowercase letters indicate significant differences among the different treatments (*p* < 0.05).

Both the chemical and biological fumigation treatments promoted the seedlings’ growth, with the Br and Ra treatments promoting the seedlings’ growth better than the Ta treatment. The LN, Br, and Ra treatments significantly promoted the stem diameter, fresh weight, and dry weight of the eggplant plants, while the Ta treatment significantly promoted stem diameter and fresh weight (Figure 7A).

The yield statistics showed that the LN treatment was the most effective in increasing the eggplant yield, while the Br treatment was the second most effective. Compared with the Br treatment, the yield of the LN treatment increased significantly by 5.01%, while the yield of the Ra, Ta, and CR treatments decreased significantly by 2.94%, 7.80%, and 3.80%, respectively. Compared with the Ta treatment, the yield of the LN, Br, Ra, and CR treatments significantly increased by 13.89%, 8.46%, 5.27%, and 4.33%, respectively. In summary, mustard, a plant of the cruciferous brassica family, can effectively replace chemical fumigation as a biological fumigation material in some situations (Figure 7B).

**Figure 8:**
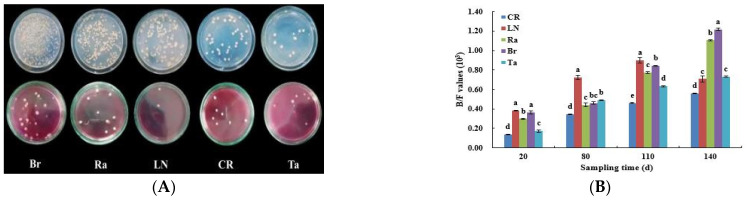
The soil’s biological characteristics during the cropping period of eggplant under different soil fumigation treatments. (**A**) The culturable bacteria colonies (above) and culturable fungi colonies (below) in soil samples collected at 110 d (in the fruiting stage); (**B**) the culturable bacteria/culturable fungi (B/F values). Different lowercase letters indicate significant differences among the different treatments (*p* < 0.05).

Fumigation treatments had a sustained effect on the biology of the eggplant soil. The treatments produced differential changes in the number of culturable bacteria and fungi during eggplant growth, with the highest number of culturable bacteria under the Br treatment and the lowest under the Ta treatment during the fruiting stage of the eggplant. The highest number of culturable fungi was found under the Br treatment and the lowest under the LN treatment (Figure 8A). As mentioned before, the ratio of the number of culturable bacteria/the number of culturable fungi (B/F value) can be used as a reliable indicator for evaluations of the soil quality, and a rise in this indicator signals an improvement in the soil quality [20]. Overall, throughout the reproductive period (20–110 d after planting), the LN treatment had the highest B/F values, followed by the Br treatment, and the Ta treatment had the lowest B/F values compared with the CR treatment. The three biological fumigation treatments showed a general trend of decreasing B/F values compared with the LN treatment (except at 140 d after planting). At 140 d after planting, the B/F value in the soil was significantly higher after the Br treatment, by 71.83%, 9.91%, 67.12%, and 117.86% compared with the LN, Ra, Ta, and CR treatments, respectively (Figure 8B). This suggests that fumigation with the cruciferous brassica mustard during the later stages of the eggplants’ growth resulted in significant differences in soil quality, which promoted plant growth and increased the yields.

## 4. Discussion

### 4.1. Diversity and Similarity of Microorganisms’ Structures

After the LN treatment, the chemical substances almost completely decomposed, and very few chemical fumigant residues remained in the soil; therefore, the soil’s physicochemical properties under the LN treatment were similar to those under the CR treatment. Consequently, the microorganisms gradually recovered and the community structure was similar to that under the CR treatment. Among the three biological fumigation treatments, both mustard and radish belong to the brassica family, and they contain glucosinolates and produce isothiocyanate compounds, which are effective ingredients for the disinfection of the soil [5]; therefore, the microbial community’s structure was similar between them. However, marigolds belong to the Asteraceae, which contain unique chemicals that are different from those of brassica. It has been reported that the chemical composition of marigolds includes thiophenes, essential oils, pigments, flavonoids, and glycosides; among these, thiophenes are characteristic secondary products and have a variety of biological activities. Among the thiophenes, α-trithiophene is one of the most representative substances with the highest content, and such substances are toxic [10,11]. The data provide a reasonable explanation for why the marigold treatment formed a unique microbial community. In addition, due to an increase in the soil organic matter content caused by the biological fumigation treatment (Table 1), the microbial communities were different between the LN and CR treatments.

The results also showed that the alpha diversity of the fungi increased under the chemical fumigation treatment LN and under the biological fumigation treatments Br, Ra, and Ta. The increased bacteria alpha diversity under the Br and Ra treatments, and the enriched soil organic matter content caused by the biological fumigation treatments, are important principles for promoting soil function compared with non-fumigation treatments. The soil’s biodiversity is sensitive to land management practices, such as planting systems, tillage, and fertilization [30]. Under the LN treatment, the diversity of both fungi and bacteria increased, which may be because of the relatively clean microenvironment caused by chemical fumigation, and it was advantageous for the soil microbial community’s rapid recovery. Numerous studies have shown that the enrichment of the soil organic matter content is beneficial for microbial accumulation. Therefore, in this study, the most important reason for the bacterial and fungal diversity increasing under fumigation treatments using cruciferous plants (Br and Ra treatments) was the enriched soil organic matter content resulting from the plant residues added to the soil. It is very interesting that, under similar situations (the addition of marigold residues to the soil and the enrichment of soil organic matter content), under the Ta treatment, the bacterial diversity decreased by 1.54%, 2.36%, and 2.26% compared with the CR, LN, and Br treatments, respectively, which indicated the deterioration and loss of function in the soil’s biological environment. This result may be due to some special toxic substances in marigold, such as thiophene isolated from marigold and other species, which is phototoxic to bacteria and fungi [31]. In addition, the EC decreased under the Ta treatment, which means that the loss of mineral ions and insufficient mineral nutrients in the soil might be another reason for the loss of the microorganisms’ diversity under this treatment. The EC represents the level of soluble salt content in the soil, and it was in the normal range for plant growth in this study. However, among the three biological fumigation treatments, the EC under the Ta treatment was the lowest, and the EC under the Br treatment was the highest, which was 1.61 times higher than the Ta treatment (Table 1). Marigold is a plant with higher levels of growth, as the growth period is longer than that of cruciferous plants [19]. In this study, the marigold had grown for about 3 weeks longer than the mustard and radish. Therefore, we believe that marigold might have absorbed more mineral ions from the soil than the cruciferous plants, which led to the EC sharply decreasing and insufficiencies in mineral nutrients and the loss of soil functions. The negative effect on the soil function caused by the Ta treatment was very great. The eggplant yield under the Ta treatment was not only lower than that under the Br and Ra treatments (cruciferous plant treatments), but was also significantly lower, by 4.16%, than the non-fumigation treatment (Figure 7B). The RDA of the soil’s physical and chemical properties and microbial communities supported this view. The soil’s physical and chemical properties had a greater influence on the formation of bacterial communities than on the fungal communities (53.36% and 45.33%, respectively), and the soil organic matter and electrical conductivity, alkaline phosphatase, etc., played much more important roles in the structure of the microorganisms’ community (Figure 3A,C).

The B/F values were used as an important index for evaluating the soil’s biological properties [32,33,34], and higher B/F values mean higher soil quality. This study showed that both chemical and biological fumigation affected the soil quality for all the growth periods of eggplant, and the results of analyzing the B/F values (Figure 8) supported the results on the eggplants’ plant growth and yield (Figure 7).

### 4.2. Biological Indicators for Transformation of the Microorganic Structure under Biological Fumigation Treatments

We were excited to find out that, at the phylum level, Rozellomycota and Olpidiomycota were the indicator species for the process of transforming the soil microbial community under the Ta and Br treatments, respectively. The accumulation of Olpidiomycota played positive roles in the optimization of the microbial structure under the Br treatment. It was surprising that Rozellomycota (RA = 13.20%) significantly increased under the Ta treatment, which was 20 times higher than that under the other treatments (RA = 0.23% to 0.65%); Olpidiomycota was 5 times higher under the Br treatment than in the other treatments. Olpidiomycota is an endophytic fungal phylum which has the function of decomposing plant residues. Latescibacteria and Rokubacteria catalyze the degradation rate of litter, breaking down cellulose and chitin [35]. Other reports have proved that Olpidiomycota together with Ascomycota and Chytridiomycota can be used as indicator species for the transformation process of soil microbial communities during the conversion from dryland to paddy fields [36], and under biochar supplementation in acidic soil, with the accumulation of Olpidiomycota and the increase in microbial diversity. The oxidative stress of rapeseed (*Brassica napus*) plants was alleviated, plant growth was promoted [37], and Olpidiomycota and Ascomycetes were enriched under a fertilizer treatment [38]. In this study, both at the genus and species levels, the functional analysis of members of the phylum Olpidiomycota also supported this conclusion. In this study, we detected two species in this phylum (*O. brassicae* and *O. bornovanus*; the RA of *O. brassicae* was about 100%). In general, *O. brassicae* and *O. bornovanus* are considered to be carriers of viruses [39], which can cause significant damage to crops. However, the classification of *O. brassicae* and its closely related species is often confusing and controversial. The latest findings clarified this controversy by using the traditional morphological identification method combined with an analysis of the molecular biology. In brief, *O. brassicae* mainly infects plants of the cruciferous brassica family, but it does not act as viral vector, and it seems very unlikely to damage plants; therefore, the authors suggested that it necessary to correct the functional annotation of the species *O. brassicae* in the fungal gene bank’s database [40]. This study provides support for this suggestion. 

According to the analysis above, Olpidiomycota is considered to be a key player in nutrient acquisition, the decomposition of soil organic matter, and carbon fixation, and its appearance provides more nutrients for plant growth and promotes the material cycle in the soil ecosystem. Therefore, we concluded that it plays a crucial role in the decomposition of mustard residues after the plant had been cut into pieces and tilled into the soil under the Br treatment. In this study, Olpidiomycota was nine times higher (RA = 9.16%) under the Br treatment than that under the Ra (RA = 1.04%) treatment, which revealed that accumulation of the phylum Olpidiomycota may be one reasonable explanation for why the Br treatment was more effective and promoted the eggplants’ growth when compared with the Ra treatment. Both mustard and radish belong to the brassica family of plants, which contain thioglycosides and can produce isothiocyanate compounds, and the isothiocyanate compounds can inhabit microorganisms in the soil, so they have similar fumigating principles in the soil [5] and similar effects on the microbial community’s construction (Figure 2A,B). Olpidiomycota is an endophytic fungus which can be widely isolated from plant tissues, so we concluded that the Olpidiomycota detected in this study mainly came from plant residues used as biological fumigants, and it was more likely to colonize in mustard tissue compared with radish tissue. Endophytic fungi exist in plant tissues and grow in roots, trunks, and leaves without causing apparent disease [41,42,43,44]. In many cases, they require protection and nutrients from the host plants; in return, the fungi contribute to the host’s growth and nutrient uptake, thus ultimately promoting plant growth. Therefore, in this experiment, under the Br treatment, the enriched Olpidiomycota must have undergone continuous infestation and colonization of the subsequently planted crop (eggplant in this experiment), establishing a mutually beneficial ecosystem with the plants and ultimately promoting the growth and yield of the eggplant.

Rozellomycota comprises a huge diversity of uncultured environmental clones, with a very few of the known endoparasitic species of algae and water mold [45]. The presence of Rozellomycota in plants as a plant endophyte has also been reported [46]. In this study, Rozellomycota under the Ta treatment was 20 times higher than that under the other treatments. At present, we could not find any studies that investigated its function in the soil. Even so, we confirmed that Rozellomycota was a biomarker for the process of transforming the soil function under the Ta treatment, and further studies on Rozellomycota and its function are worthwhile. For example, as an endophytic fungus, does it have a positive role in maintaining soil function by decomposing plant residues? Is it possible to isolate its specific members and determine their function in the soil? Does the accumulation of the phylum correlate with a loss of soil function under the Ta treatment?

### 4.3. Predicted Fungal Function and Key Microbial Groups Associated with Soil Health

The results for the predicted fungal functions successfully supported our conclusion that the soil health was superior under the Br treatment compared to the Ra and Ta treatments. In this study, fumigation with mustard inhibited the growth and aggregation of pathotrophic fungi, which reduced the risk of soil-borne diseases occurring in the plants, as well as enhanced the colonization by saprophytic fungi (Figure 6A). Saprophytic fungi can decompose refractory organic matter and dead host cells in the soil, providing important energy for microbial activities. Compared with fumigation with either radish or marigold, fumigation with mustard had a significant effect on killing plant pathogens (members of the pathotrophic fungi) in the soil (Figure 6B). However, it is possible that the disinfection effect of the biological fumigation treatment was weaker when compared with chemical fumigation (Figure 6A,B).

The LEfSe analysis demonstrated some key microbial groups related to soil health, which supported the analyses of the microbial groups’ functions mentioned above. For example, under the Br treatment, the genus *Olpidium* (an important member of the phylum Olpidiomycota) was accumulated compared with the CR treatment (Figure 4B). As previously mentioned, Olpidiomycota plays a key role in microbial nutrient acquisition, the decomposition of soil organic matter, and carbon fixation. Ascomycetes are mostly saprophytic fungi that can decompose recalcitrant organic matter such as lignin and keratin, playing an important role in nutrient cycling [47]. It has been reported that Ascomycetes have strong metabolic activity, and their metabolites include terpenes, alkaloids, flavonoids, and sterols [48,49]. In this study, Microascales (an important member of the phylum Ascomycetes) were significantly enriched from order to family (Figure 4A). Therefore, we believe that the accumulation of Microascales and *Olpidium* might contribute to the improved soil health under the Br treatment. In another example, *Alternaria* was enriched under the Ta treatment when compared with the Br treatment (Figure 4D). Most of the *Alternaria* species are common plant pathogens; leaf spot and leaf litter caused by *Alternaria* can cause significant economic losses [50,51]. Therefore, *Alternaria* might play an important role in the damage to the soil health caused by the Ta treatment. The bacterial LDA showed that *Bacillus* was significantly enriched under the Br treatment (Figure 5B–D) when compared with the other three treatments (the CR, LN, and Ta treatments). Both *Bacillus* and *Pseudomonas* accumulated under the Br treatment when compared with the LN treatment (Figure 5C). *Bacillus* is a recognized genus of probiotics that can activate ineffective mineral nutrients in the soil and improve plants’ absorption and utilization efficiency, such as biological nitrogen fixation and phosphorus solubility, which are beneficial for improving the utilization of potassium fertilizer and resistance to soil-borne diseases. *Pseudomonas* is known as a beneficial bacterium for plant growth because it enhances sulfate uptake [52] and acts as an antagonist against soil pathogenic fungi [53]. Therefore, *Bacillus* and *Pseudomonas* play a key role in improving soil health by improving soil nutrient utilization and antagonizing pathogenic fungi under the Br treatment. 

Overall, according to the soil properties and the crop growth promotion, the use of the three biofumigants clearly showed the consequences of the different biological fumigation methods, where mustard was the best and radish was the second best, and marigold might negatively impact the crops. The three biofumigants significantly altered the soil’s fungal and bacterial microbial communities compared with the chemical fumigation. The LN treatment created a relatively clean soil environment, which promoted soil health by increasing the microorganisms’ alpha diversity. Among the three biological fumigation methods, the Br treatment was the best, followed by the Ra treatment, while the Ta treatment had a negative impact on the soil function, which could cause a loss in crop yields. The Br treatment was superior for improving the soil health over the Ra treatment. The microbiological principle of the Br treatment was as follows. Fumigation with mustard can effectively kill plant pathogenic fungi (such as *Alternaria*) and reduce pathotrophic fungi and plant pathogens, thereby reducing the risk of soil-borne diseases occurring. With the depletion of pathotrophic fungi, the saprophytic trophic fungi (such as Olpidiomycota, its member genera *Olpidium*, and Microascales) accumulated, which provided much more material for the colonization by microorganisms and increased the microbial diversity. In addition, fumigation with mustard can enhance soil nutrient utilization and disease resistance by accumulating some specific beneficial bacteria (such as *Bacillus* and *Pseudomonas*). But fumigation with marigold can cause serious damage to the soil function, such as nutrient deficits, the loss of soil bacterial diversity, and the accumulation of pathogenic fungi. In summary, we provided the above exposition to illustrate the differences in the soil microorganisms’ structures associated with soil function between biological fumigation with mustard (promoting soil function) and biological fumigation with marigold (damaging soil function).

In this region, marigold is planted in large areas (the flowers are harvested as drug materials), but the plant residues are left in the fields and are tilled into the soil after the flowers have been harvested, which leads to a higher possibility of damaging the soil function. Thus, it is necessary to deeply study how the toxicity occurs in the soil, and researchers should focus on the marigold plants’ secondary products and their influence on the structure of soil microorganisms. Suitable successional crops after cropping marigolds should be carefully selected, and sufficient chemical fertilizers combined with microorganic agents (or bio-organic fertilizers) should be applied to improve the soil nutrient levels and to optimize the soil’s biological structures, through which the loss of soil function might be repaired and the productivity might be partly restored.

## 5. Conclusions

Among these three biological fumigation methods, the Br treatment was the best for alleviating the crop’s CRPs, and may be a substitute for chemical fumigation in some situations. However, the Ta treatment risks losing soil quality and productivity.

The Br treatment was superior compared to the Ra treatment. The Br treatment improved the soil nutrient utilization and decreased the risk of soil-borne disease by increasing the abundance of probiotic bacteria (e.g., *Bacillus* and *Pseudomonas*), optimized the soil’s physicochemical properties (increased the organic matter content), and improved the soil’s biological properties (mainly increased the bacterial and fungal alpha diversity, decreased the abundance of pathotrophic fungi, and increased the abundance of saprotrophic fungi). Among them, the accumulation of beneficial functional microorganisms, such as Olpidiomycota and its member genera *Olpidium*, Microascales, and the probiotic bacteria *Bacillus* and *Pseudomonas*, under the Br treatment made important contributions to improving the soil health.

However, marigold caused serious damage to the soil function, such as nutrient deficits, the loss of the soil’s bacterial diversity, and the accumulation of pathogenic fungi. In light of the situation wherein marigolds are planted in large areas as an economic crop in Western China, suitable successional crops should be carefully selected, and sufficient chemical fertilizers combined with microorganic agents (or bio-organic fertilizers) should be applied to repair the loss of soil function.

## Figures and Tables

**Table 1:** The soil’s physicochemical properties and enzyme activity after the eggplant soil fumigation treatment.

Treatment	OM (g/kg)	EC (ms/cm)	WC (%)	pH	Urease (mg/g)	Alkaline Phosphatase (mg/g)	Sucrase (mg/g)	Catalase (mg/g)
CR	12.34 ± 0.20 c	515 ± 6.06 b	11.9 ± 0.0002 ab	7.52 ± 0.02 a	0.98 ± 0.00 a	2.23 ± 0.06 b	1.47 ± 0.03 b	1.74 ± 0.01 a
LN	11.31 ± 0.76 c	495 ± 3.21 bc	12.2 ± 0.0036 a	5.86 ± 0.02 c	1.06 ± 0.03 a	2.49 ± 0.16 a	1.67 ± 0.10 a	1.49 ± 0.20 ab
Ra	18.42 ± 0.51 ab	483 ± 3.84 c	12.7 ± 0.0029 a	7.55 ± 0.01 a	1.05 ± 0.02 a	2.29 ± 0.21 b	1.59 ± 0.05 ab	1.25 ± 0.04 b
Br	20.27 ± 0.65 a	556 ± 6.35 a	12.4 ± 0.0030 a	7.43 ± 0.01 b	0.95 ± 0.00 a	2.28 ± 0.09 b	1.69 ± 0.01 a	1.26 ± 0.05 b
Ta	17.07 ± 1.16 b	345 ± 5.49 d	11.2 ± 0.0019 b	7.54 ± 0.00 a	0.98 ± 0.16 a	2.48 ± 0.01 a	1.68 ± 0.00 a	1.54 ± 0.02 ab

Note: OM, EC, and WC represent the organic matter, electrical conductivity, and water content, respectively. Different lowercase letters indicate significant differences among the different treatments (*p* < 0.05).

**Table 2:** Dominant phyla of fungi and bacteria after the eggplant soil fumigation treatment (*p* < 0.05, average RA > 1%).

Treatment	CR	LN	Ra	Br	Ta
Fungi					
p__Ascomycota	54.87% ab	47.75% b	66.99% a	49.42% b	49.76% b
p__Mortierellomycota	15.61% ab	19.18% a	10.19% b	11.69% ab	11.17% b
p__Rozellomycota	0.23% b	0.53% b	0.61% b	0.65% b	13.20% a
p__Olpidiomycota	0.07% b	1.80% b	1.04% b	9.16% a	1.14% b
Bacteria					
p__Acidobacteria	29.69% ab	29.5% ab	23.72% b	27.6% ab	35.33% a
p__Chloroflexi	15.15% a	13.21% ab	12.75% ab	12.14% bc	10.36% c
p__Gemmatimonadetes	7.25% b	7.87% ab	8.52% ab	8.92% a	6.22% c
p__Actinobacteria	7.41% a	6.92% ab	5.71% bc	4.94% c	4.67% c
p__Planctomycetes	1.45% b	2.14% ab	2.37% a	2.56% a	2.32% a
p__Nitrospirae	2.22% a	2.09% ab	1.57% c	1.7% b	2.07% ab
p__Firmicutes	0.99% bc	0.7% c	2.71% a	1.9% ab	0.46% c
p__Parcubacteria	0.81% bc	1.31% ab	1.6% a	1.72% a	0.7% c
p__Saccharibacteria	0.9% b	0.77% b	1.07% ab	1.18% ab	1.35% a
p__Latescibacteria	0.85% ab	1.15% ab	0.77% b	1.07% ab	1.34% a

Note: Different lowercase letters indicate significant differences among the different treatments (*p* < 0.05).

## Data Availability

The sequences obtained in this study have been submitted to the NCBI Sequence Read Archive (SRA) under the Bioproject ID PRJNA1139406.

## Supplementary Materials

The following supporting information can be downloaded at: https://www.mdpi.com/article/10.3390/microorganisms12102044/s1, Figure S1: A and B are biofumigants of cruciferous brassica; C is the figure of laminated fumigation, D is a figure of the film uncovered for drying, and marigold was widely cultivated as medicinal plant in the region; Figure S2: 18 guilds according to the basic three trophic types.
