# Supplementary material for: Soil Microorganism Interactions under Biological Fumigations Compared with Chemical Fumigation
Source: Microorganisms. 2024 Oct 10;12(10):2044. doi: 10.3390/microorganisms12102044 (PMC11509853; doi:10.3390/microorganisms12102044)

Supplementary Figure S1: A and B are biofumigants of cruciferous brassica; C is the figure of laminated fumigation, D is a figure of the film uncovered for drying, and marigold was widely cultivated as medicinal plant in the region.

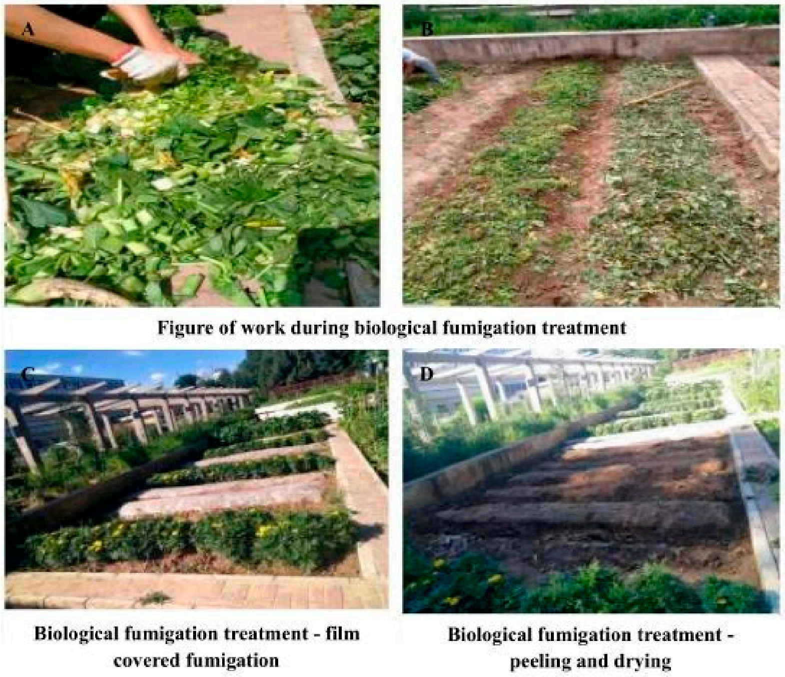

Supplementary Figure S2: 18 guilds according to the basic three trophic types.

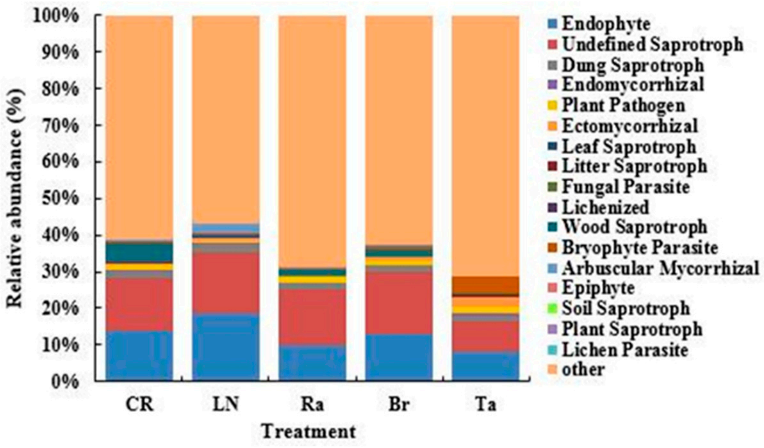

Supplement: Supplementary file 1 [file microorganisms-12-02044-s001.zip › microorganisms-3217772-supplementary.pdf]
